# The Initial Inflammatory Response to Bioactive Implants Is Characterized by NETosis

**DOI:** 10.1371/journal.pone.0121359

**Published:** 2015-03-23

**Authors:** Ljubomir Vitkov, Wolf-Dietrich Krautgartner, Astrid Obermayer, Walter Stoiber, Matthias Hannig, Michaela Klappacher, Dominik Hartl

**Affiliations:** 1 Department of Zoological Structure Research, Cell Biology, University of Salzburg, Salzburg, Austria; 2 Clinic of Operative Dentistry, Periodontology and Preventive Dentistry, Saarland University, Homburg, Germany; 3 Children’s Hospital and Interdisciplinary Center for Infectious Diseases, University of Tuebingen, Tuebingen, Germany; The Hospital for Sick Children and The University of Toronto, CANADA

## Abstract

Implants trigger an inflammatory response, which is important for osseointegration. Here we studied neutrophil extracellular trap (NET) release of human neutrophils in response to sandblasted large-grit acid etched (SLA) implants using fluorescent, confocal laser scanning and scanning electron microscopy. Our studies demonstrate that human neutrophils rapidly adhered to SLA surfaces, which triggered histone citrullination and NET release. Further studies showed that albumin or acetylsalicylic acid had no significant effects on the inflammatory response to SLA surfaces. In contrast to bioinert materials, which do not osseointegrate, the bioactivity of SLA surfaces is coupled with the ability to release NETs. Further investigations are necessary for clarifying the role of NETosis for osseointegration.

## Introduction

Endosteal implants are sterile foreign bodies surgically inserted into bone with an associated inflammatory host’s response [1–3]. In cases of bioinert implants, a soft tissue encapsulation takes place. When bioactive implants are used, a direct interface forms between bone and implants, i.e. osseointegration is achieved [1]. However, the intimate mechanisms of osseointegration and the differences between the initial responses to bioinert and bioactive implants remain poorly defined.

In the initial stage of osseointegration, spaces around titanium implants are filled with blood coagulum infiltrated with leukocytes [2,3] Polymorphonuclear neutrophils, (PMNs) are rapidly recruited to sites of inflammation and have been shown to attach within minutes to artificial implant surfaces [4–6], triggering the production of reactive oxygen species (ROS) [7–9]. Besides these findings, the fate and functionalities of human PMNs in response to implants remained poorly understood. Upon infection and inflammation, PMNs expel their own DNA, a process termed ‘neutrophil extracellular trap’ (NET) formation or ‘NETosis’, since the major form of NET formation is associated with PMN cell death [10]. NETs are efficient in limitation of microbial spreading before sufficient quantities of leukocytes are recruited to the endangered area [10]. However, NETs can also be triggered by non-infectious agents [10,11] or placental microparticles [11] and can be harmful for the host [12–19]. Mechanistically, the production of ROS [20] and the citrullination of histones [21,22] have been closely linked to NETosis. In addition to PMNs, thrombocytes are also recruited to sites of inflammation and have been shown previously to adhere to titanium surfaces [6,23,24]. Regarding PMN activation, platelets have been described to drive NET formation through a mechanism involving Toll-like receptor 4 (TLR4) [16,17,25]. Based on these studies, we hypothesized that PMNs undergo NETosis in response to implants.

For this purpose, we comprehensively studied (i) whether PMNs and thrombocytes attach to SLA and poly-D-lysine-coated surfaces, (ii) whether NETosis occurs on such surfaces, and if so, (iii) whether platelets, albumins, acetylsalicylic acid (ASA) and IgG contribute to implant-induced NETosis. Therefore, we quantified the responses of PMNs and platelets after contact with SLA surfaces of standardised titanium samples in a short-term incubation system. Our studies demonstrate that PMNs form NETs in response to SLA implants. Understanding this NET release by bioactive SLA implants might contribute to new possibilities for modulating osseointegration.

## Materials and Methods

### Blood sampling and titanium plate preparation

Healthy, one month unmedicated volunteers without chronic diseases (n = 4, two male and two female, aged between 25 and 39 years) were selected for donation of capillary blood. All blood donors gave written consent to take part in the study. These studies were approved by the ethical committee of the University of Tuebingen. Blood sampling from each volunteer was performed in two sessions with an interval of at least one week. Capillary blood was collected under sterile conditions from finger pricks using safety lancets and processed [26,27]. Samples were directly transmitted from the fingertips of volunteers to the plates. In this way, the initial stage of osseointegration was imitated as spaces around titanium implants are filled with blood coagulum [1,2]. The direct blood sample transition also prevents the contamination with endotoxin [26,27]. The plates were produced from medical use titanium (ASTM F67 (DIN 3.7065), containing 0.35% F, 0.1% C, 0.05% N, 0.35% O and 0.013% H) with the same sandblasted large-grit acid etched (SLA) surface finish as used for SLA Octagon implants (Dental Ratio, Düsseldorf, Germany). All plate surfaces were additionally UV sterilized for 1 hour prior to further use. Blood attachment to the SLA surface was examined on three sets of differently pre-treated plates: (i) uncoated, (ii) albumin-coated, and (iii) albumin/ASA coated. For coating, plates were immersed in the respective solutions for 10 minutes (Table 1). To prepare surface attachment samples, these plates were covered with fresh, untreated capillary blood immediately after coating and incubated in a humid chamber at 37°C and 5% CO_2_ for 4 hours. The same procedure was applied to the set of uncoated (control) plates. Subsequently, the bulk of coagulated blood was removed from the SLA surfaces with the help of fine tipped forceps, thus leaving only the directly surface-attached layer in place. The plates were then rinsed in phosphate buffered saline (PBS) and fixed in 4% paraformaldehyde in PBS. For immunocytochemistry-based quantification of PMNs and NETs, two samples on SLA plates were prepared per subject, session and pre-treatment regime (Table 1), resulting in a total of 12 plates per pre-treatment from 4 subjects. For the assessment of platelets by immunocytochemistry and SEM, additional 4-hour-incubated samples on SLA plates, and also on uncoated or poly-D-lysine-coated glass cover slips, were taken in at least one of the sessions (2–3 per subject in each case). To test possible platelet disintegration early in the clotting process, further plates (again 2–3 per subject) were already fixed after 5 min of incubation.

**Table 1:** Surface treatment on SLA titanium plate sets.

Set	Label	surface type	pre-treatment
1	C	control (untreated SLA)	none
2	ALB	Albumin-coated SLA	5% human albumin in 0.9% NaCl
3	ASA	albumin/ASA-coated SLA	5% human albumin and 1 mM acetylsalicylic acid in 0.9%NaCl

### Immunocytochemistry

For unambiguous identification of PMNs and for the detection of platelets, immunofluorescence staining was performed as follows: Samples were washed in phosphate buffered saline (PBS, pH 7.4), blocked with 10% normal goat serum in PBS containing 10mM glycine and 0.2% Tween 20, and then incubated with primary antisera against appropriate molecular markers. Antisera against citrullinated histone H3 (mouse anti-human CitH3, cit R2+R8+R17 IgG1, Abcam ab80256; 1:100) and neutrophil elastase (rabbit anti-human NE IgG, Abcam ab21595; 1:50) were employed for detection of PMNs, rat anti-human CD41 IgG1 (553847, BD Pharmingen, 1:100) for detection of platelets. Goat anti-rabbit DyLight 488 (Abcam ab96883; 1:100), goat anti-mouse TRITC (ab6786, Abcam, 1:100) and goat anti-rat Alexa Fluor 488 (A11006, Invitrogen, 1:1000) were applied as secondary antibodies. DNA was stained with DAPI (Sigma Aldrich) or propidium iodide (P4170, Sigma Aldrich, Germany). Stained plates were mounted on glass slides and coverslipped using GelMount (BioMeda) mounting medium, and viewed and photographed in a Reichert Polyvar microscope equipped for fluorescence microscopy.

### Quantification of PMNs and NETs

For photo sampling, groups of 30 randomised non-overlapping photos per plate were taken along two parallel transects across the entire surface, thus enabling adequate representation of the central and peripheral areas. Image stacks of each group were created and analysed using the software *ImageJ*. Cell numbers per stack were assessed using the cell counter plugin. PMNs were identified by positive staining for NE, nucleated but NE-negative cells were classified as ‘other cells’. For the quantification of NETs, the outlines of areas covered by extracellular DNA were traced and the total area per stack was measured in μm^2^. The software SPSS 20 was used for data processing and statistical analysis. The three types of surface treatment (untreated, albumin-coated, albumin/ASA-coated) were compared by univariate analysis of variance with subject and sampling session as covariates, and by Tukey HSD post hoc test.

### Confocal laser scanning microscopy (CLSM)

CLSM was employed to support qualitative screening for the presence of platelets. Samples on poly-D-lysine-coated cover slips, either incubated for 4 h, or only for 5 min, and immuno-stained for CD41 as described above, were analysed and photographed in a Zeiss LSM 510 meta UV CLSM (Carl Zeiss GmbH, Vienna, Austria).

### Scanning electron microscopy (SEM)

SEM served to detect NETs and platelets. PFA-fixed samples on SLA plates and glass cover slips were, dehydrated in a graded series of ethanols, critical point dried and sputter-coated with gold, and finally viewed in a ESEM XL30 (FEI Company, Philips, Eindhoven, Netherlands) scanning electron microscope operating at 25 kV.

## Results

### Neutrophil adhesion

**Fig 1:**
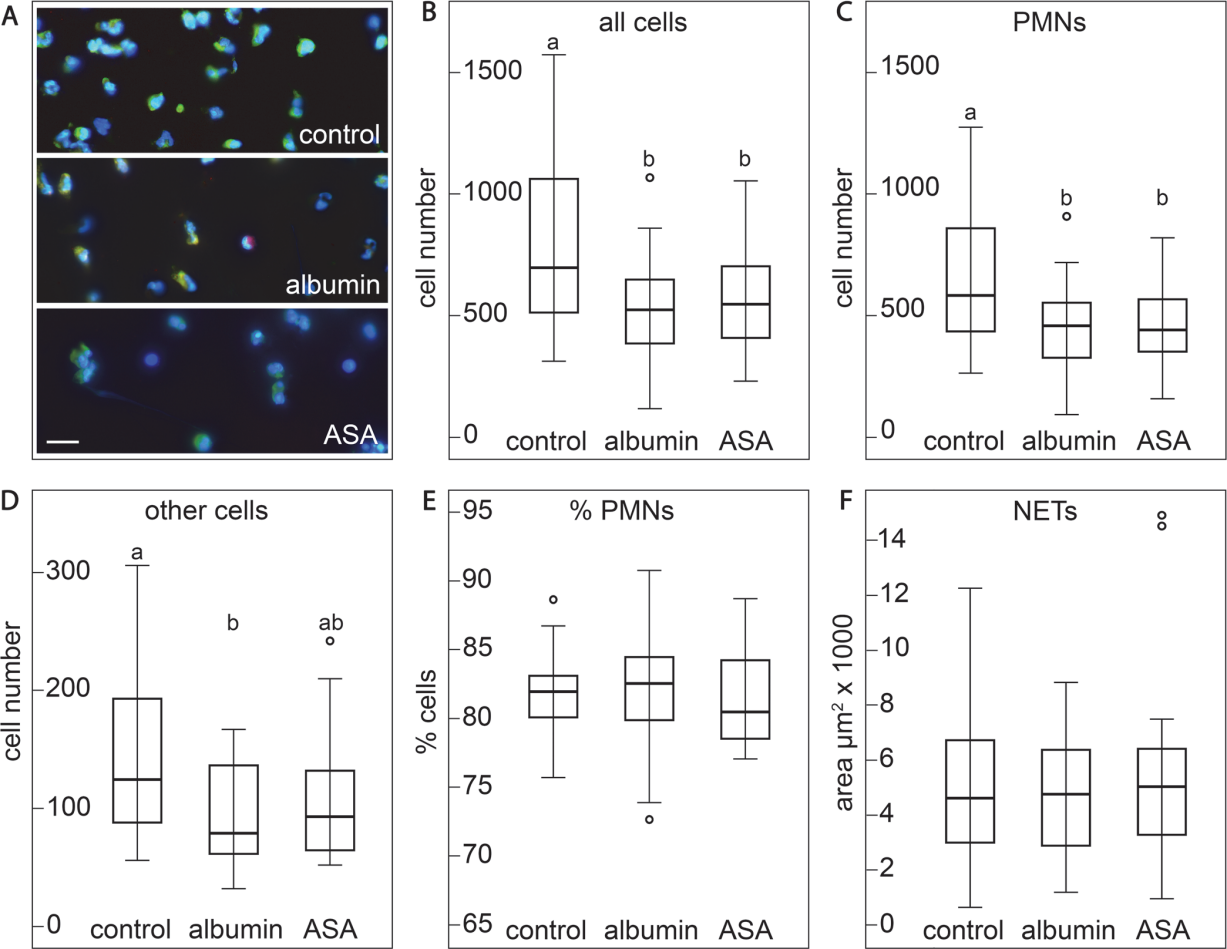
Cell adhesion to SLA surfaces from whole peripheral blood with different pre-treatments. (A) Representative images of the three pre-treatment groups (blue: DAPI, green: NE, red: CitH3), scale bar: 20μm. (B-F) Boxplots (interquartile range; line: median, whiskers: 1.5 x interquartile range) showing absolute (B-D) and relative (E) cell numbers and the areas covered by NETs (F) for the three groups. Superscript letters indicate groups of statistically significant differences. The three types of treatment were compared by univariate analysis of variance and by Tukey HSD post hoc test with subject and sampling session as covariates,. Superscript letters (a, b) indicate groups of statistically significant differences at the *P*<0.05 level (similar letters: no significant differences, different letters: significant difference).

**Fig 2:**
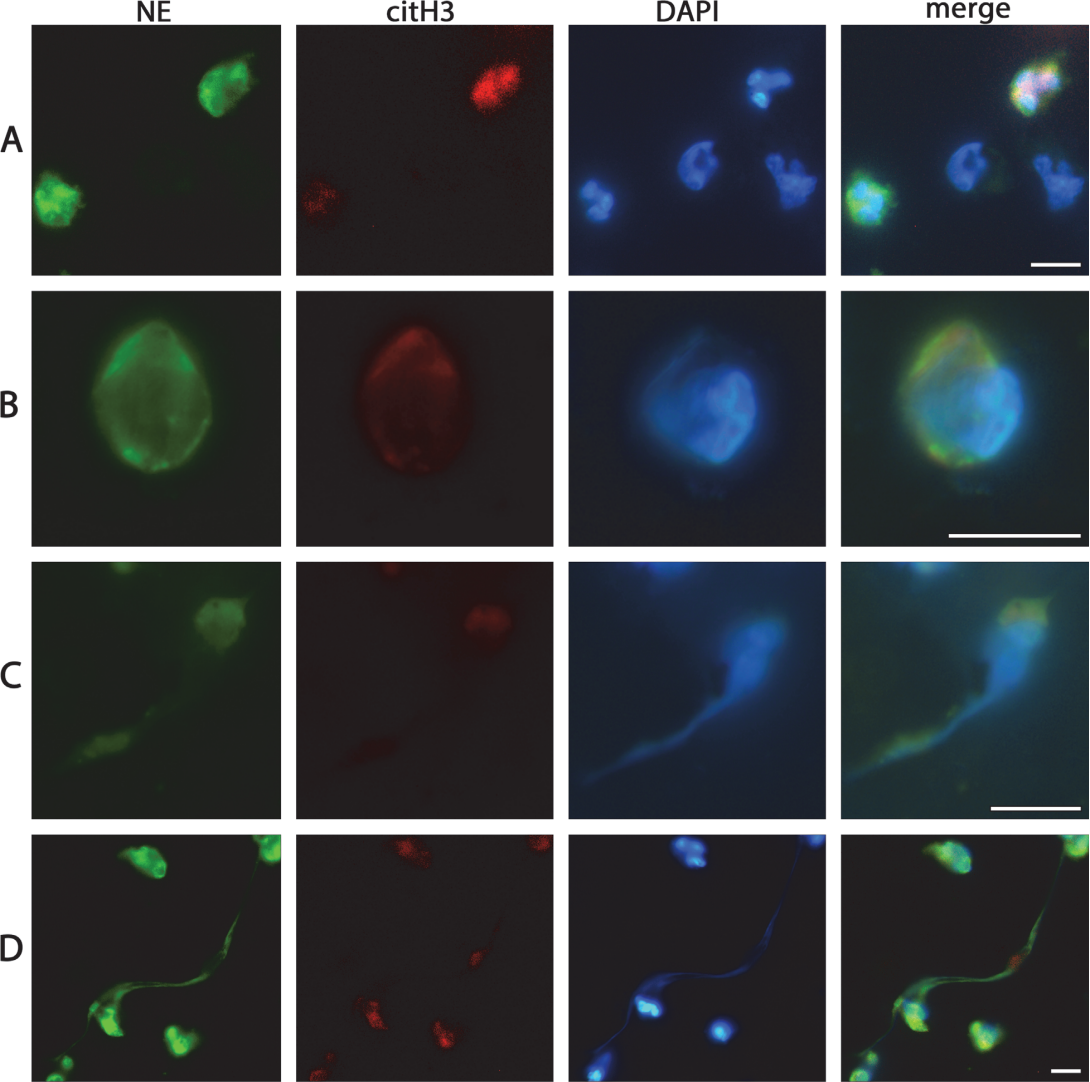
Characteristics of cells adhered to SLA surface from whole peripheral blood as detected by immunofluorescence. (A) NE-postitive PMNs with their typically lobulated nuclei, and other NE-negative nucleated cells. (B) A single PMN committed to the NETotic cascade as clearly shown by decompensated chromatin, the swollen, partly disrupted nucleus and NE and citH3 staining co-located with chromatin and at cytoplasmic locations. (C) Chromatin extrusion from PMN. (D) Fully spread NETs between PMNs of different activation states. Scale bars: 10μm.

**Fig 3:**
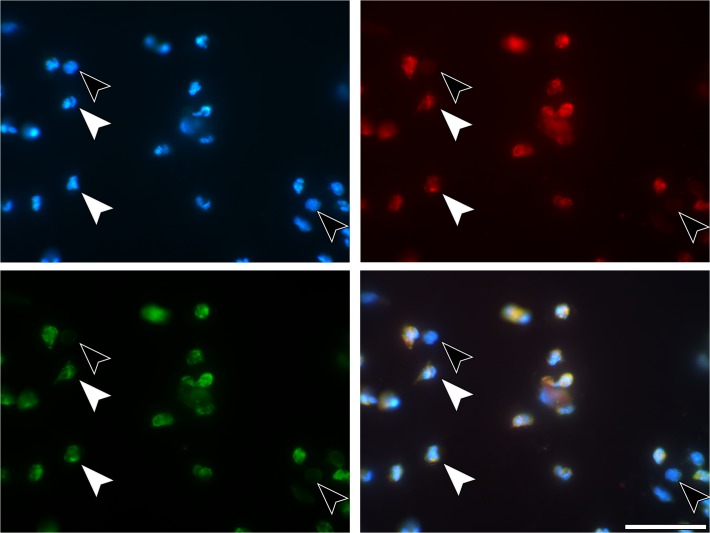
Immunostaining of neutrophils. (A) 4h SLA sample. PMNs (white arrows) und others, not further determined, cells (black arrows). Blue—DNA staining with DAPI, (B) red—immunostaining for CitH3, (C) immunostaining for neutrophil elastase, (D) merged. The other cells lack both NE and CitH3. Scale bars: 50μm.

Examination of blood cell attachment to the surfaces of the SLA titanium plates under the fluorescence microscope revealed that after 4 hours of incubation, several types of nucleated cells had attached to the titanium surfaces of the plates of all three pre-treatment schedules (untreated, albumin-coated, albumin/ASA-coated) (Fig. 1A, S1 Fig., Table 1). The majority of the attached cells (80.8% ± 5.4%–82.4% ± 5.1%) were PMNs as identified by immunolabelling for NE (Fig. 1B-E, Fig. 2A, Fig. 3). Although there was no difference between pre-treatment schedules in regard to the relative amounts of PMNs (Fig. 1E), total cell numbers were higher on the surfaces of the uncoated control plates than on those of the two coated sets of plates (P < 0.05). This suggested that a similar trend toward enhanced cell attachment to uncoated SLA surfaces also exists for NE-negative cells. However, for these cells, a statistically significant difference (P < 0.05, Fig. 1B,C) was only found between the uncoated and the albumin-coated plates (Fig. 1B-E). SLA plates incubated for 5 min showed only a scarce presence of nucleated cells, a finding contrasting to those incubated for 4 h. This difference strongly indicated that the PMN transmigration from the bulk coagulum to the SLA surface is a lasting process and suggests that the heterogeneity of NETotic stages of PMNs may be a consequence of their diverse time-points of interacting with the SLA surface.

### NETosis

**Fig 4:**
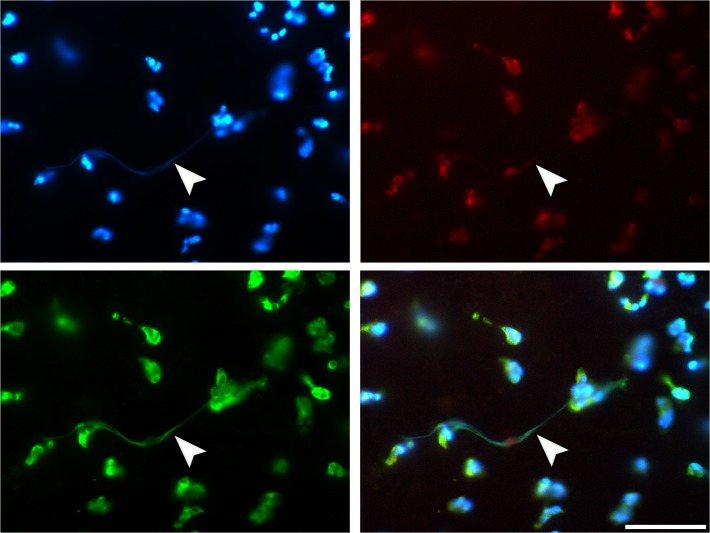
Immunostaining of neutrophils with NETs. 4h SLA sample. Staining as in Fig. 3. Overview: ripe NETs (arrows) between the adhered cells. Scale bars: 50μm.

**Fig 5:**
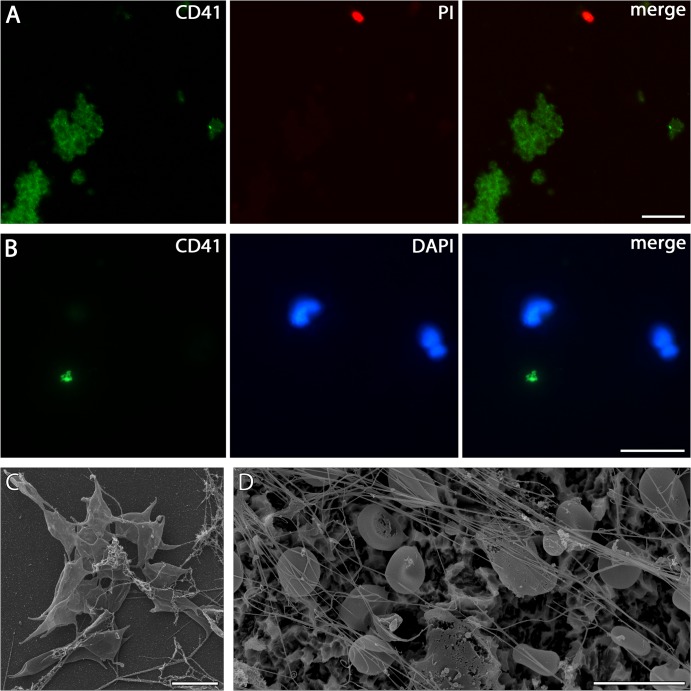
Immunostaining of platelets (A) Detection of platelets adhered from whole peripheral blood to PDL-coated cover slips incubated for 5 min by immunolabelling against CD41 (green). Large clusters of cells can be found accompanied by few individual nucleated cells (red—PI). Scale bar 10 μm. (B) Platelets staining for CD41 (green) on SLA surface incubated for 5 min with whole peripheral blood are scarcely present between adhered PMNs as shown by lobulated nuclei (blue—DAPI). Scale bar 20 μm. (C) Platelets shown by SEM on uncoated glass cover slips incubated for 4 h with whole peripheral blood. Scale bar 5 μm. (D): SLA incubated for 4 h with whole peripheral blood show no platelets, but numerous erythrocytes as well as fine fibres with fibrin-like and NET-like morphology. Scale bar 10 μm.

A subset of PMNs attached to the SLA surface displayed diverse stages of NETosis as defined by previous studies [20,28]. Accordingly, such cells stained positive for citrullinated histone H3 (citH3) and showed swollen nuclei, a characteristic pattern of chromatin decondensation, and—in some cases—also chromatin extrusion (Fig. 2A-D, Fig. 4). In addition to these NETotic PMNs, the SLA surfaces were also decorated with fully spread NETs. These are in all relevant morphological aspects similar to those of NETs from other in vitro and from in vivo sources, as documented in previous studies on NETosis [10,29]. The areas covered by NETs showed large variation between the individual samples and did not significantly differ between the three pre-treatment regimes. All qualitative morphological findings on SLA surface attached NETs made by fluorescence microscopy were supplemented by SEM analysis (Fig. 5D). In contrast, no signs of NETs were observed on the poly-D-lysine-coated surface (Fig. 5B). We did not observed any necrotic and any apoptotic PMNs.

### Platelets

In order to test a possible interaction of platelet and PMN adhesion, the SLA surfaces of subsets of plates from three pre-treatment regimes were screened for the presence of these characteristic disc-shaped cell fragments. This was done by fluorescence microscopy after immunostaining for CD41, and on separately prepared specimens under the SEM (see Methods). After four hours of incubation, neither of these two methods succeeded in detecting the presence of platelets (Table 2). Given this negative result, supplementary analyses by fluorescence microscopy, SEM and CLSM were made using samples on SLA plates incubated for only 5 min, and samples on uncoated and poly-D-lysine-coated glass cover slips incubated for 5 min or 4h. This was performed in order to test whether the absence of platelets on the 4-hour-incubated plates was due to quantitative disintegration during the incubation time, or rather due to (as yet undetermined) properties of the SLA titanium surface. SLA plates incubated for 5 min showed, in contrast to those incubated for 4 h, a scarce but noticeable presence of platelets (Fig. 5B), while more CD41 positive platelet aggregates could be found on poly-D-lysine-coated and uncoated glass coverslips, particularly with those after 5 min incubation (Fig. 5A). The presence of such platelet-aggregates on 4-hour-incubated uncoated cover slips was also confirmed by SEM (Fig. 5C; Table 2).

**Table 2:** Presence of platelets on test surfaces as detected by different microscopic methods.

Surface		detection method	5 min	4 h
SLA		SEM	±	-
		FM	±	-
glass coverslips glass coverslips	Uncoated	SEM	no data	+
	poly-D-lysine	CLSM	++	+

- not detectable

± scarcely present

+ present

++ abundantly present.

SEM: scanning electron microscopy; FM: fluorescent microscopy, CLSM confocal laser scanning microscopy.

## Discussion

The inflammatory host response to bioactive implants is poorly understood, particularly the role of PMNs. Here we demonstrate that human neutrophils rapidly adhered to SLA surfaces and release extracellular DNA structures with characteristics of NETs. This finding also suggests that some NET components might be beneficial for osseointegration, supported by the inability of bioinert materials to osseointegrate at all [1] and by the deterioration of osseointegration through administering anti-inflammatory drugs during the initial stages of osseointegration [30–32].

Activated platelets can induce NETosis [16,17,25] and treatment with ASA, a platelet inhibitor, has been described to decrease NET formation [16]. As a systematic pre-medication with ASA is in general not feasible in dental implant surgery because of the risk of bleeding, we used the ASA ability to bind to albumin up to 85% [33,34] in order to apply it topically on the albumin-coated implant surface. This pattern of ASA application is surgically feasible, as the ASA effects remain restricted closely to the implant surface. Despite the ability of thrombocytes [6,23,24] to attach to titanium surface, the amount of SLA-adhered thrombocytes was not significant und cells were completely lysed after four hours. The treatment with ASA did also not affect the inflammatory response to the SLA surface. Thus, the lack of efficiency of the ASA treatment strongly suggests that platelets are not involved in triggering NETosis on the SLA surface in our experimental system.

Platelet lysis through ROS production has been previously reported [35,36] and the strong respiratory burst on implants surfaces [7–9] as well as NETosis on SLA surface suggest that ROS may be responsible for platelet lysis. Therefore, one may speculate that the NETosis/thrombocyte relationship on SLA surface may differ from NETosis/thrombocyte relationships within blood vessels, where NETosis and thrombocytes are closely associated and even activated thrombocytes have been described to induce NETosis [16,17,25].

Our findings concerning the neutrophil adhesion are in accordance with earlier reports concerning the neutrophil response to both smooth titanium [7–9] and other artificial implant surfaces [4–6]. However, PMNs adhered to SLA surface show diverse stages of NETosis, characterized by swollen nuclei and chromatin alteration [20,22] as well as completely spread NETs. In contrast, neither full NETs nor initial NETosis stages were evident on poly-D-lysine-coated surfaces. Histone citrullination indicates PMNs activation and represents the initial stage of NETosis on SLA [21,22]. In our studies, histone citrullination characteristics support the notion that the vast majority of PMNs attached to SLA titanium surface undergoes NETosis. A main trigger of the inflammatory response to smooth artificial surfaces is the adsorbed IgG [9,37,38]. Pre-coating the artificial surfaces with albumin or fibrinogen disables the adhesion of other proteins including IgG and essentially attenuates both PMN adhesion and inflammatory response on smooth artificial surfaces [37–40]. The pre-coating of smooth implant surfaces with human IgG has resulted in considerable higher levels of cell activation [37–40]. In contrast, the albumin pre-coating of SLA surface in our experiments slightly attenuated PMN adhesion, but had no significant effect on NETosis. These findings suggest that PMN Fc-gamma-receptors may somewhat contribute to the adhesion to SLA surfaces, but do not influence the NETosis initiation on them. Consequently, another inflammatory stimulus, considerable stronger than the non-self-recognition by IgG and/or complement, appears to play the main role for the inflammatory response to the SLA surfacein our experimental system.

SLA surfaces are micro/nano-textured TiO_2_-coated titanium surfaces, which accelerate the osseointegration in comparison with the smooth titanium surfaces [41–43]. Indeed, the distinct morphological characteristic of bioactivity of endosteal implants is their micro/nano-textured surface [44,45]. Interestingly, micro- and nano-sized TiO_2_ particles have been demonstrated to activate human PMNs [46] *in vitro* and to cause a pronounced inflammatory reaction in animal models marked by neutrophilia [47–49]. By virtue of the surface similarity, SLA surfaces might comply similarly to micro- and nano-sized TiO_2_ particles. Indeed, both SLA surfaces and nano-particles [50] are characterised by pronounced protein citrullination. The SLA surface properties are a possible explication of the difference [51] between PMN inflammatory responses to smooth artificial surfaces [37–40] and SLA ones.

Collectively, our results demonstrate that the initial inflammatory response to bioactive implants is marked by NETosis, which is not the case for bioinert poly-D-lysine-coated surfaces. NETosis is the first reported mark of initial response to a bioactive implant, so far as the bioactivity of endosteal implants has been defined only as the ability to osseointegrate. The deterioration of osseointegration through administering anti-inflammatory drugs during the initial stages of osseointegration [30–32] and the ability of statins to enhance both NETosis [52] and osseointegration [53–55] also suggest that distinct components of the inflammatory response to bioactive implants might be beneficial for the osseointegration. The *in vivo* role of NETosis for osseointegration remains to be defined in future studies.

## Supporting Information

S1 Dataset Rare data of proband 1. (XLS)

S2 Dataset Rare data of proband 2. (XLS)

S3 Dataset Rare data of proband 3. (XLS)

S4 Dataset Rare data of proband 4. (XLS)

S1 Fig SLA surface. Overview of the SLA micro/nano-textured surface. Scale bars: 5μm.
(TIF)
