# Supplementary figures and images for: The Initial Inflammatory Response to Bioactive Implants Is Characterized by NETosis
Source: PLoS One. 2015 Mar 23;10(3):e0121359. doi: 10.1371/journal.pone.0121359 (PMC4370506; doi:10.1371/journal.pone.0121359)

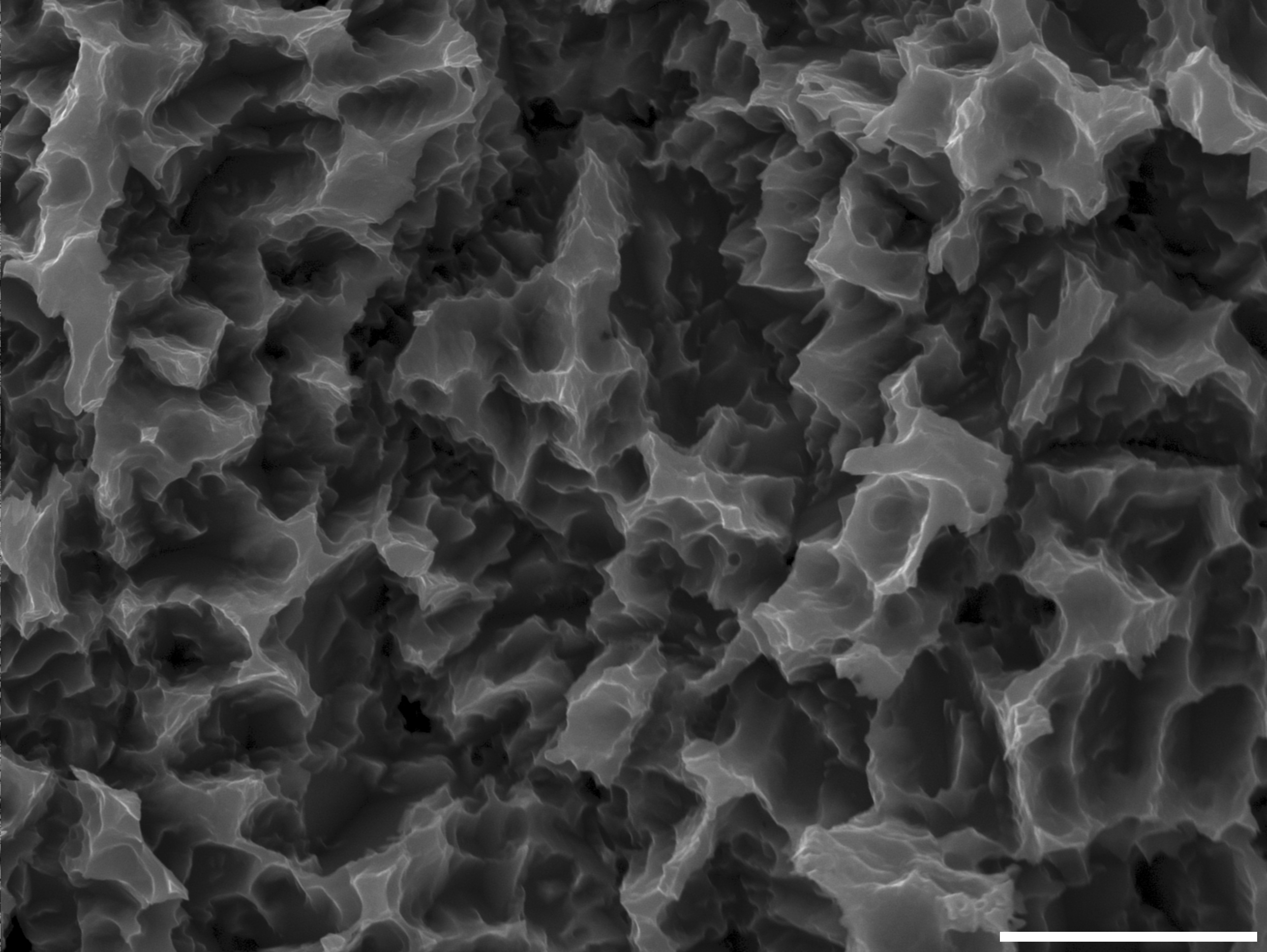

Supplement: S1 Fig — Overview of the SLA micro/nano-textured surface. Scale bars: 5μm. (TIF) [file pone.0121359.s005.tif]
